# Update on Color Flow Imaging in Obstetrics

**DOI:** 10.3390/life12020226

**Published:** 2022-01-31

**Authors:** Kwok-yin Leung, Yung-Liang Wan

**Affiliations:** 1 Gleneagles Hospital Hong Kong, Hong Kong, China; ky@kyleung.org; 2 Department of Medical Imaging and Intervention, Linkou Chang Gung Memorial Hospital, College of Medicine, Chang Gung University, Taoyuan 333423, Taiwan

**Keywords:** 3D/4D ultrasound, STIC, color Doppler, obstetrics, radiant flow, superb microvascular imaging

## Abstract

Color flow imaging (CFI), being non-invasive, is commonly used in obstetrics to study the fetal and placental circulations. The conventional CFI modes include color Doppler flow imaging (CDFI) and power Doppler imaging (PDI). In recent years, there is increasing use of new modes, including high-definition flow imaging (HDFI), radiant flow, microvascular flow imaging (MVFI), and three-/four-dimensional rendering in glass-body mode. Compared to CDFI, HDFI can show a higher resolution and sensitivity and allow the detection of slower flows. MVFI increases the sensitivity to fine or low-flow vessels while producing little or no motion artifacts. Radiant flow shows the blood flow with a sense of depth and reduces blood overflow. Glass-body mode, showing both gray-scale and color-flow information, can demonstrate the heart-cycle-related flow events and the vessel spatial relationship. In this review, the characteristics and applications of the various CFI modes in obstetrics are discussed. In particular, how these new technologies are integrated in detailed diagnostic and early morphology scans is presented.

## 1. Introduction

Color flow imaging (CFI), being non-invasive, is commonly used in obstetrics to study the fetal and placental circulations. CFI can be used to identify the presence and direction of blood flow, assess the anatomy of the fetal heart and peripheral vessels, and provide a beam/vessel angle correction for a proper measurement of velocity [1]. The addition of CFI to gray-scale ultrasonography can improve the prenatal diagnosis of, among others, heart defects, umbilical cord abnormalities, and placental disorders [2,3,4,5,6,7]. The conventional CFI modes include color Doppler flow imaging (CDFI) and power Doppler imaging (PDI). In recent years, there is an increasing use of new modes, including high-definition flow imaging (HDFI), microvascular flow imaging (MVFI), radiant flow, and three-/four-dimensional rendering in glass-body mode. The objective of this review is to summarize the characteristics of the various CFI modes and to evaluate their use in obstetrics. In particular, how these new technologies are integrated into detailed diagnostic and early morphology scans, as recently recommended [8,9,10,11,12,13], is discussed.

## 2. Color Flow Imaging Modes

**Figure 1:**
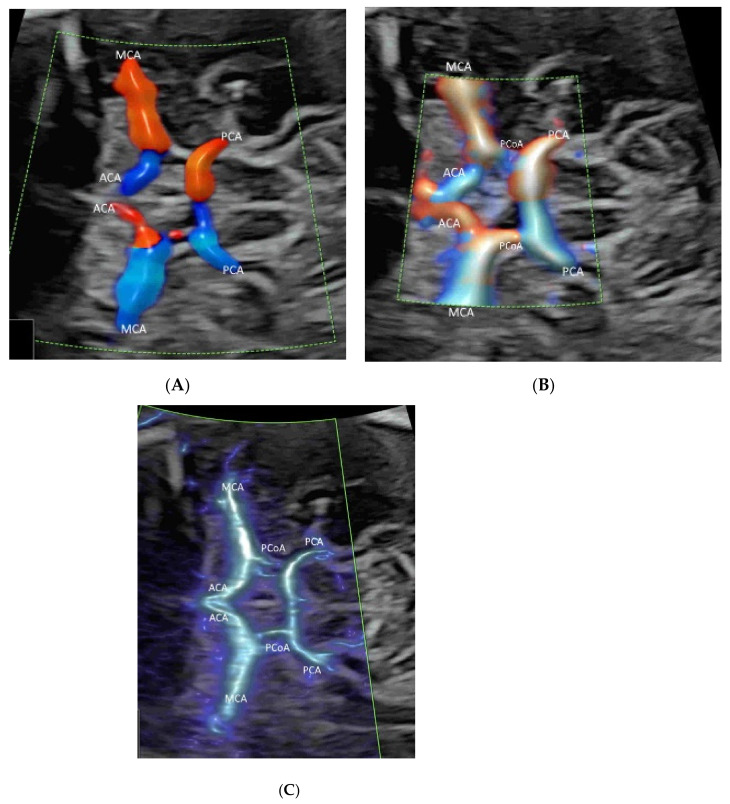
Demonstration of the Circle of Willis (COW) in a transverse plane of a normal fetus at 25 weeks’ gestation by color Doppler flow imaging (CDFI) (**A**), high-definition flow imaging (HDFI) (**B**), and microvascular flow imaging (MVFI) (**C**) with radiant flow. Note that the anterior cerebral artery (ACA), the middle cerebral artery (MCA), and the posterior cerebral artery (PCA) are all well demonstrated in all three modes, while the posterior communicating artery (PCoA) is demonstrated in HDFI (**B**) and MVFI (**C**) but not CDFI (**A**).

Compared to CDFI, which measures the frequency shift generated by the velocity of flow of the red blood cells within a vessel (Figure 1A), PDI measures the amplitude (power) of the Doppler signal generated by their signal intensity. By calculating the difference in the intensity of luminance without consideration of the direction and the velocity, PDI does not have signal aliasing and can allow detection of flows with smaller velocities [1]. However, PDI does not provide directional information of blood flow and is unable to demonstrate the presence or absence of turbulence. 

High-definition flow imaging (HDFI), also called bi-directional power Doppler imaging, utilizes a digital broadband assessment of Doppler signals, which combines the Doppler frequency shifts with signal amplitudes. HDFI assigns a color based on the measured Doppler shift, without taking into account the direction or velocity [14,15]. Compared to CDFI, HDFI can show a higher resolution and sensitivity (Figure 1B). However, CDFI, PDI, and HDFI are affected by clutter and motion artifacts, which are derived from pregnant women’s motion, pulsation, and respiration [16]. To remove these artifacts, a single-dimensional wall filter is usually applied. Unfortunately, this filter also removes overlapping low-velocity flow signals, resulting in the loss of the low-flow components [16]. 

Microvascular flow imaging (MVFI) is a new technology which applies a multi-dimensional or a highly sophisticated wall filtering system to separate low-velocity flow signals from the overlapping clutter artifacts [17,18,19,20]. As such, MVFI increases the sensitivity to fine or low-flow vessels while producing little or no motion artifacts (Figure 1C). Besides, MVFI uses high-frequency sampling techniques and a high frame rate, displaying images with a high resolution [17,18,19,20]. MVFI can detect flow, even when the insonation angle is suboptimal. Some newly developed MVFI systems are capable of providing color-coded directional information superimposed on the B-mode image. Similar technology has been developed by several industries, including Superb Microvascular Imaging (SMI) technology (TOSHIBA Inc., Tokyo, Japan), MicroFlow imaging (Philips Healthcare), MV-Flow (Samsung Medison), and SlowFlowHD (GE Healthcare).

**Figure 2:**
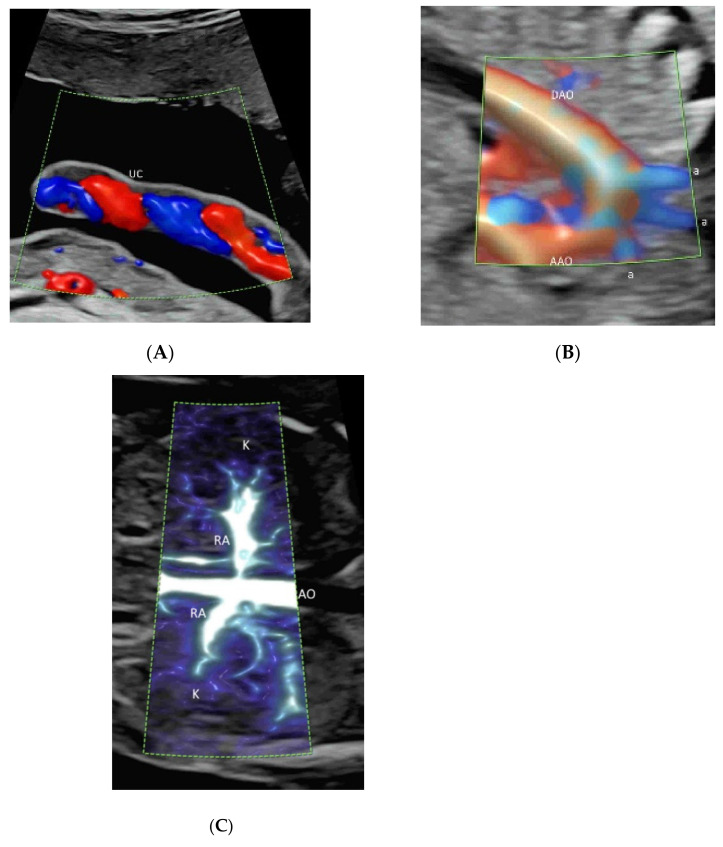
Demonstration of radiant flow applied to color Doppler (**A**), high-definition (**B**), and microvascular(**C**) flow imaging. Note that the blood flow is shown with a sense of depth in the umbilical cord (UC) vessels (**A**), the aortic arch and its branches (a) (**B**), and the renal arteries (RA) and their branches (**C**). AAO, ascending aorta; DAO, descending aorta; AO, aorta.

Radiant flow (GE Healthcare) or LumiFlow (Samsung Medison) is a new technology which converts the index of erythrocyte density in a certain area into a height index and can be applied to CDFI, PDI, HDFI, or MVFI [21] (Figure 2A–C). This new two-dimensional (2D) technology shows the blood flow with a sense of depth or a “three-dimensional (3D)-like” appearance, reduces blood overflow, and enhances the spatial comprehension of blood vessels and their boundaries [21].

**Figure 3:**
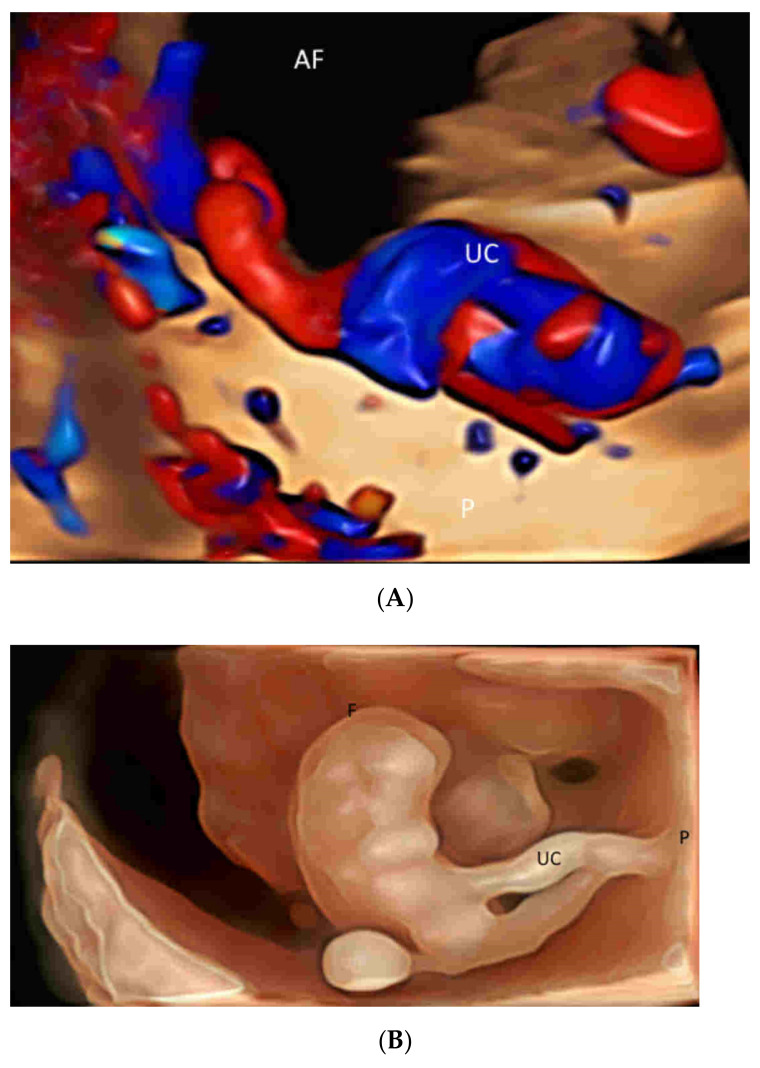
(**A**) Spatio-temporal Image Correlation (STIC) volume acquisition, displayed in the glass-body mode with both gray-scale and color information, demonstrates the umbilical cord (UC) insertion into the placenta (P). AF, amniotic fluid. (**B**) Three-dimensional ultrasound, displayed in the HDlive silhouette mode, demonstrates the umbilical cord (UC) connecting a normal fetus (F) to the placenta (P) at 8 weeks’ gestation.

The newly developed glass-body mode, after a 3D or 4D spatio-temporal image correlation (STIC) volume acquisition, demonstrates the heart cycle-related flow events and vessel spatial relationship by displaying both gray-scale and color information [4,22] (Figure 3A). Besides, a special software allows the adjustment of a light source to achieve lighting and shadowing effects and, hence, enhance the depth perception and spatial impression of the vascular structures [4]. HDlive silhouette, another special mode, displays blood flow, blood vessel walls, and their semitransparent lumens with enhanced clarity [23] (Figure 3B).

## 3. Safety and Optimization

Diagnostic obstetric ultrasonography is generally considered safe [1]. Particular attention should be paid when CFI is used because the energy output is higher with CFI than that with B-mode. It is important to limit fetal exposure to CFI to as low as reasonably achievable (ALARA), especially during the first trimester. Both the thermal index and mechanical index should be kept at ≤1.0 [1]. 

To optimize image quality, the selection of the correct ultrasound equipment, an appropriate route of examination (transabdominal or transvaginal), presets, and CFI modes is required [1,8]. It is important to keep the region of interest (ROI) as small as possible by narrowing the scanning angle, increasing the imaging depth, and then applying magnification CFI [1]. To avoid motion artifacts, ultrasound examinations are performed during fetal quiescence and without maternal movement. For MVFI, reducing acoustic output and gain can reduce flash artifacts [20]. An appropriate CFI mode should be selected to study different circulations. CDFI, PDI/HDFI, and MVFI can allow the detection of rapid, intermediate, and slow flows, respectively (Figure 1B and Figure 2A,C). 

## 4. Clinical Applications in Obstetrics

CFI is commonly used to evaluate the anatomy and blood flow of the fetal heart, the peripheral vessels, umbilical cord, and the placenta [1,2,3,4,5,6,7]. CFI is also helpful in the evaluation of various structures in the first trimester and in twin pregnancies [9,24].

### 4.1. Fetal Heart 

The ISUOG encourages adding CFI in routine mid-trimester screening [13]. CFI may facilitate imaging of the various cardiac structures, especially in obese women [25], and aid in the detection of heart defects, such as pulmonary or aortic stenosis with abnormal blood flow patterns [5,26]. A transverse sweep through the heart from the four-chamber view, through outflow tracts to the three-vessel trachea view is helpful in the assessment [13]. The acquired clip can facilitate a subsequent frame-by-frame review of the ultrasound images. Judicious use of the cine-loop function can partly overcome the effects of motion artifacts [1]. 

**Figure 4:**
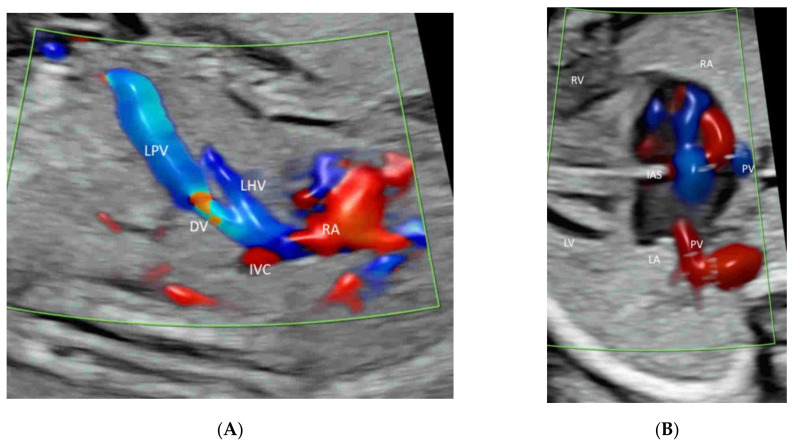

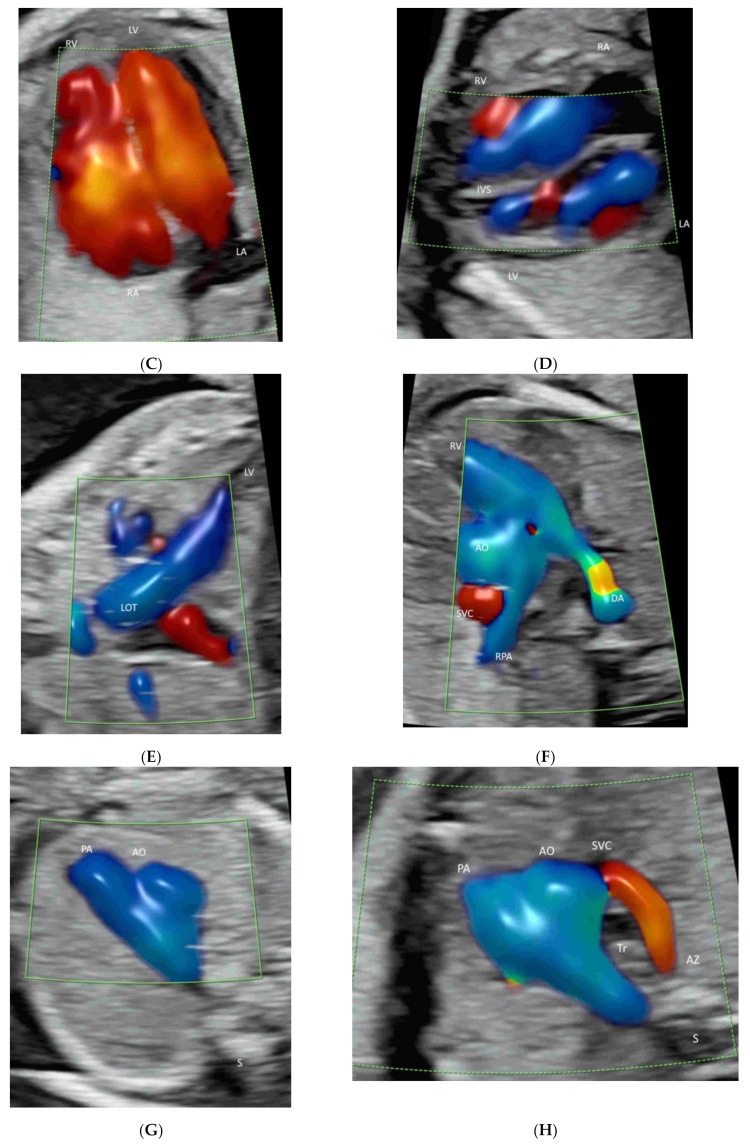

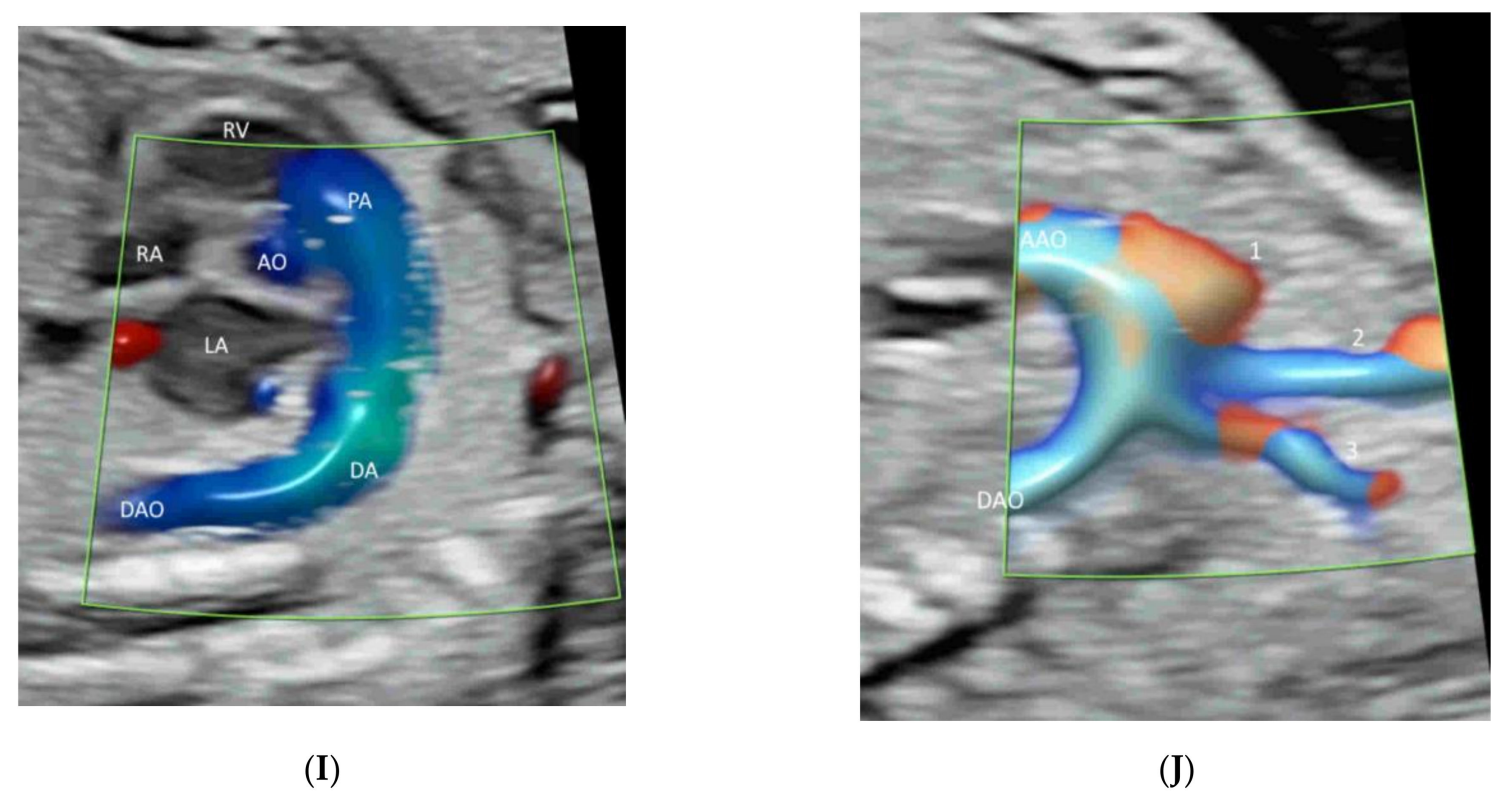
Color flow imaging in fetal echocardiography: Ductus venosus (DV) and inferior vena cava (IVC) (**A**), pulmonary veins (PV), atrial septum and foramen ovale (**B**), atrioventricular valves (**C**), ventricular septum (**D**), semilunar valves (**E**–**H**), ductal arch (**I**), and (g) aortic arch (**J**). Note the left ventricular outflow tract (LOT) in (**E**), branches of RPA in (**F**), three-vessel view in (**G**), three-vessel trachea view in (**H**), and the three ascending branches (1–3) in (**J**). LPV, left portal vein; LHV, left hepatic vein; RA, right atrium, LA, left atrium; RV, right ventricle; LV, left ventricle; IAS, interatrial septum; IVS, interventricular septum; DA, ductus arteriosus; PA, pulmonary artery; RPA, right pulmonary artery; Tr, trachea, AZ, azygous vein; AO, aorta; DAO, descending aorta; AAO, ascending aorta.

The ISUOG recommends adding CFI in fetal echocardiography [13]. The AIUM recommends using CFI to evaluate (a) the systemic veins (including superior and inferior venae cavae and ductus venosus), (b) pulmonary veins (at least two: one right vein and one left vein), (c) atrial septum and foramen ovale, (d) atrioventricular valves, (e) ventricular septum, (f) semilunar valves, (g) ductal arch, and (g) aortic arch [11] (Figure 4A–J). Radiant flow shows detailed intracardiac blood flow information [21]. HDFI is useful to detect pulmonary veins [27]. 

Four-dimensional fetal echocardiography may help in assessing cases of complex heart defects, including conotruncal malformations, aortic arch abnormalities, and abnormal pulmonary venous return [28,29,30,31]. Using color Doppler with STIC in the glass-body mode can show normal and abnormal anatomy of the fetal heart and great vessels [4]. In particular, the relationship of the great vessels in heart defects can be demonstrated. HDlive flow and/or HDlive flow silhouette can be used to examine various heart defects by revealing the spatial relationships among the fetal cardiac structures [23]. Three-dimensional rendered images are useful for parental counseling to parents, and STIC volume can facilitate teleconsultation [21]. 

### 4.2. Fetal Brain

**Figure 5:**
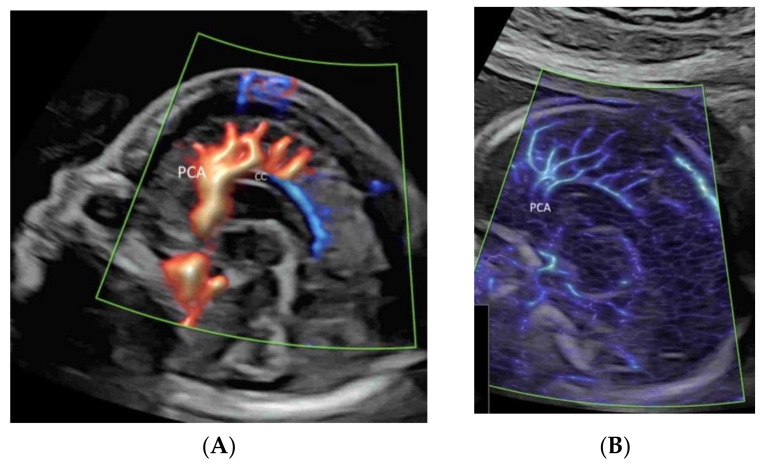
Demonstration of the pericallosal artery (PCA) and its branches in a mid-sagittal plane of two normal fetuses at 21–23 weeks’ gestation by high-definition flow imaging (**A**) and microvascular flow imaging (**B**). Note a normal course of the PCA around the corpus callosum (CC).

**Figure 6:**
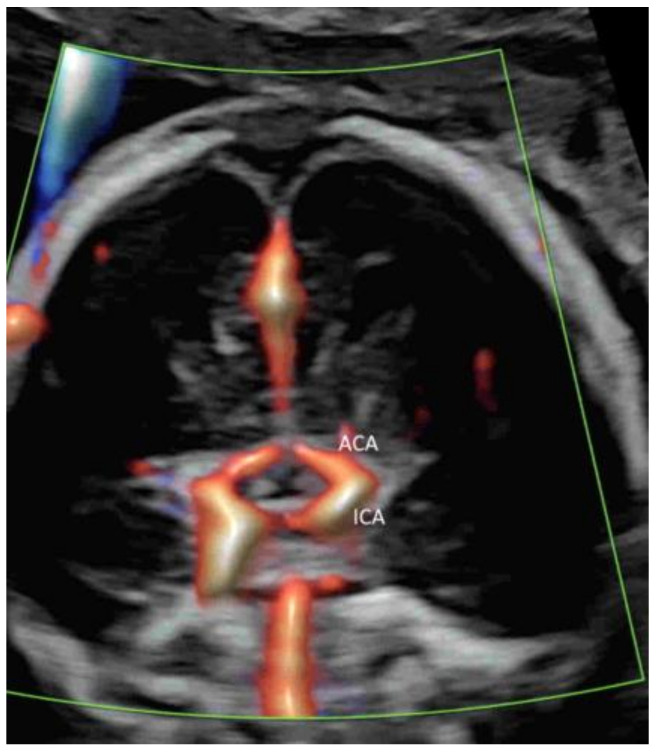
Coronal view in high-definition flow through the anterior fontanelle of a normal fetus at 20 weeks’ gestation showing the optic chiasm, an X-shaped structure at the center, surrounded by the supracavernous segment of the internal carotid artery (ICA) and the anterior cerebral artery (ACA).

**Figure 7:**
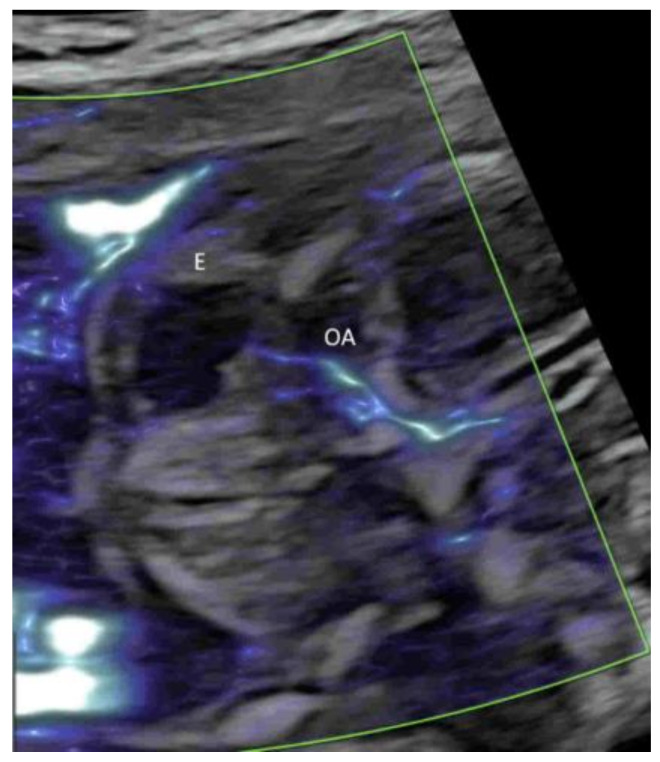
Microvascular flow imaging in a transverse view of a normal fetal brain at 20 weeks’ gestation showing the ophthalmic artery (OA). E, eye.

Applying PDI, HDFI, or MVFI in the mid-sagittal plane can demonstrate the anterior cerebral artery and pericallosal arteries with their branches (Figure 5A,B). However, its role is marginal in the assessment of the corpus callosum, according to the ISUOG guidelines [12]. In the coronal plane through the thalamus, the blood vessels around the optic chiasm can be seen close to the cranial base and in the midline (Figure 6) [12]. CDFI, HDFI, and MVFI can show the circle of Willis in the transverse plane with different degrees of details (Figure 1A–C). Transvaginal high-resolution 3D ultrasound with HDFI can demonstrate medullary vessels of white matter [32]. Using MVFI in the transverse plane can show slow-blood flow in the small intracranial vessels and orbital vessels (Figure 7) [33]. The assessment of brain vascularization may help with diagnosis and predict neurological prognosis in some central nervous system conditions [32]. Two-dimensional and three-dimensional CDFI/HDFI are effective tools to diagnose and characterize the vein of Galen aneurysmal malformation [34]. 

### 4.3. Fetal Abdomen and Other Fetal Vessels

**Figure 8:**
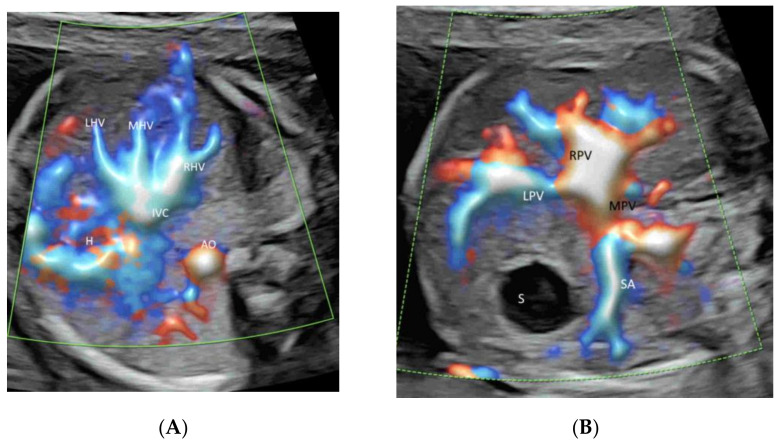
High-definition flow imaging of precordial venous system in two transverse planes of a normal fetal abdomen at 22 weeks’ gestation. An image of the upper fetal abdomen shows the normal ‘trident sign’ of the left (LHV), middle (MHV), and right (RHV) hepatic veins connecting to the inferior vena cava (IVC) (**A**). Note the connections of the left portal vein (LPV) and the main portal vein (MPV) to the right portal vein (RPV) at the stomach level in (**B**). S, stomach; aorta (Ao), splenic artery (SA) and stomach (St).

A targeted examination of the fetal umbilical-portal venous system is needed when an abnormality in the venous system is found or suspected while measuring fetal abdominal circumference or when a heart defect or other fetal anomaly is found [35]. A systematic assessment of this venous system can be achieved using CDFI or HDFI in two transverse planes and one sagittal plane, as suggested by Yagel [36] (Figure 4A and Figure 8A,B). Such assessment can facilitate the prenatal diagnosis of venous abnormalities [36]. The use of HDFI and radiant flow aids the prenatal diagnosis of an aberrant course of ductus venosus [37]. 

**Figure 9:**
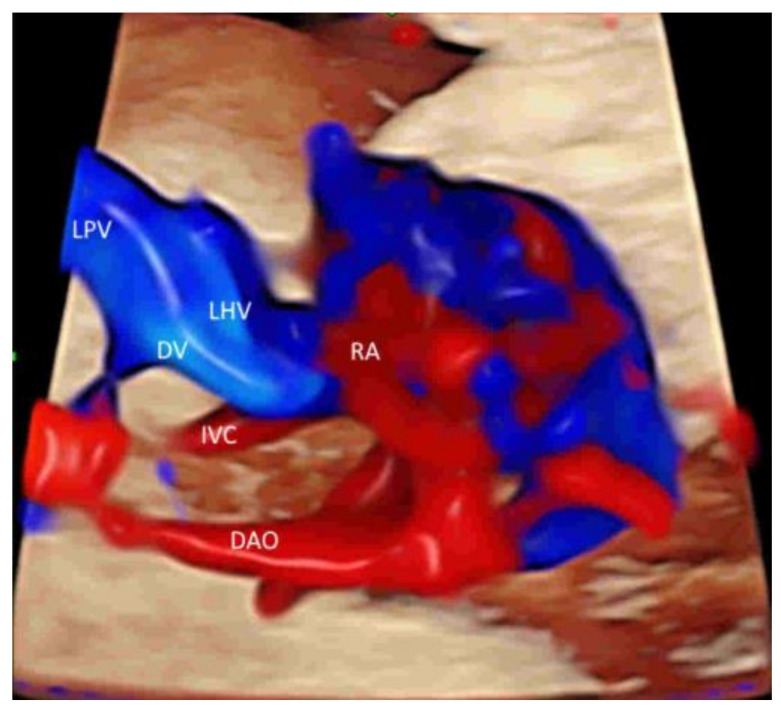
Spatiotemporal image correlation volume acquisition in color Doppler displayed in glass-body mode in a longitudinal view of a normal fetus at 20 weeks’ gestation showing the umbilical vein (UV), the left portal vein (LPV), the left hepatic vein (LHV), the ductus venosus (DV), and the inferior vena cava (IVC). Note that the ductus venosus (DV), which arises from the left portal vein (LPV), left hepatic vein (LHV), and inferior vena cava (IVC), connects with the subdiaphragmatic vestibulum. Right atrium (RA), descending aorta (DAO).

Leung KY recently described a new approach of using STIC with rendered volume in glass-body mode and TUI to facilitate the assessment of this complex venous system [38] (Figure 9). The connection of the hepatic veins, ductus venosus, and inferior vena cava to the fetal heart and their spatial relationships can be displayed in glass-body mode. STIC cine-loop can show blood flow of the precordial venous system in a cardiac cycle with two different pathways to the right atrium [38]. Further studies are required to assess if this approach can improve the prenatal diagnosis of venous abnormalities.

**Figure 10:**
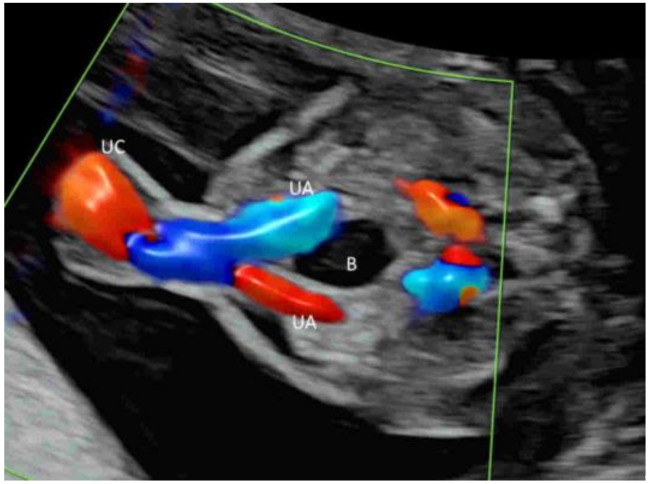
Color Doppler in a transverse plane of a normal fetus at 20 weeks’ gestation showing two umbilical arteries (UA) surrounding the urinary bladder (B). UC, umbilical cord.

To assess the number of cord vessels, it is a common practice to use CFI with low flow settings to identify the two umbilical arteries that surround the urinary bladder and then are directed towards the cord insertion (Figure 10) [39]. This can facilitate the prenatal diagnosis of a single umbilical artery.

**Figure 11:**
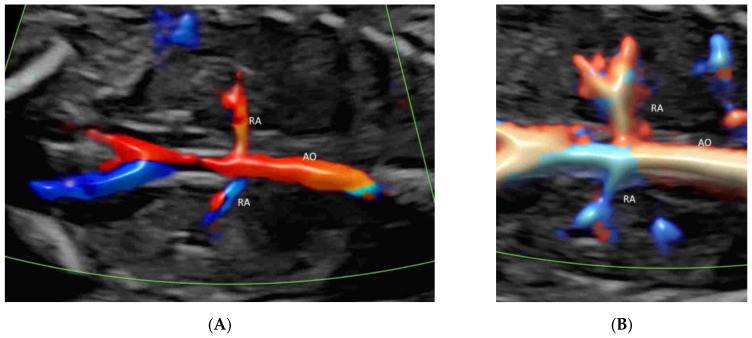
Color Doppler (**A**) and high-definition (**B**) flow imaging in a coronal plane through the back of a normal fetus at 20 weeks’ gestation showing both renal arteries (RA) arising from the aorta (AO).

In a coronal plane through the fetal back, just anterior to the spine, CFI with low flow settings shows both renal arteries and their extension into the renal pelvis (Figure 2C and Figure 11A,B) [39]. MVFI shows vessels from the branches of the renal artery to peripheral small blood capillaries (Figure 2C) [33]. MVFI can be used to confirm the absence of blood flow to a multicystic dysplastic kidney or an absent kidney [40].

**Figure 12:**
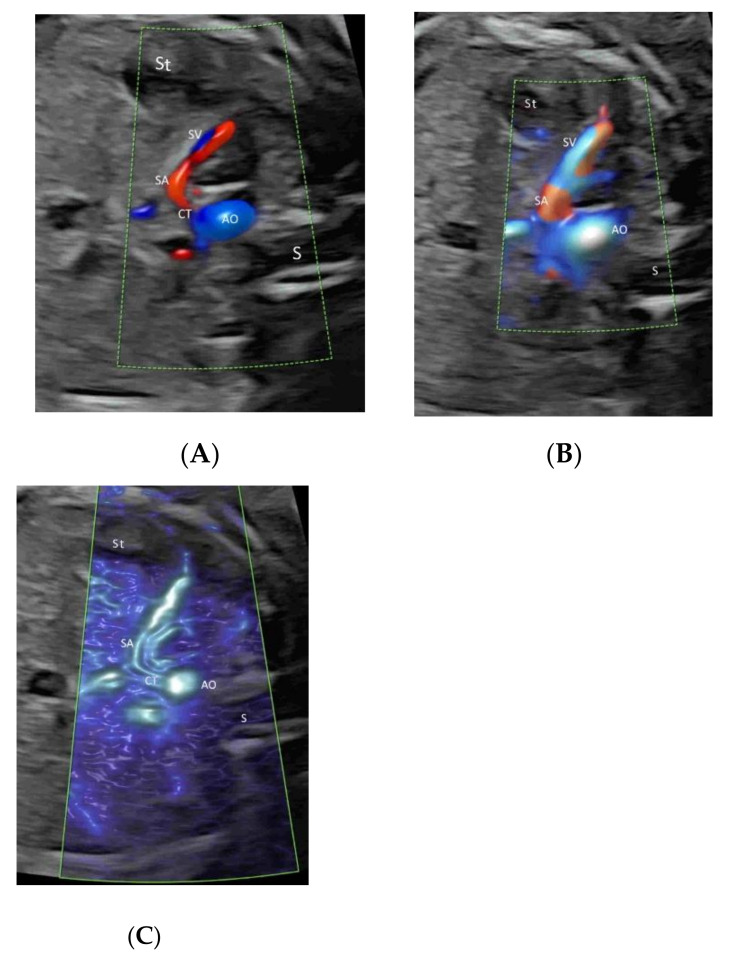
Color Doppler (**A**), high-definition (**B**) and microvascular (**C**) flow imaging in a transverse plane of a normal fetus at 23 weeks’ gestation showing the splenic artery (SA), a branch of the celiac trunk (CT) arising from the aorta (AO). St, stomach; S, spine.

HDFI or MVFI can be used selectively in the fetal lung, liver, spleen, adrenal gland, and limbs when an abnormality is found or suspected in these structures (Figure 12A–C) [40]. In fetuses affected by a congenital diaphragmatic hernia, PDI/ HDFI can be used to study segments of the pulmonary arteries in the lung contralateral to the hernia, and an altered pulmonary PDI/HDFI at 26 to 38 weeks is associated with neonatal mortality [41]. CFI of the splenic artery can help in the prenatal diagnosis of heterotaxic syndromes [42]. 

CDFI or HDFI can show fetal vascular tumors, such as hepatic hemangiomas, limb vascular tumors, and sacrococcygeal teratomas [43,44]. When a superficial cystic structure over a limb or body is found on ultrasonography, CFI is useful to assess the presence and intensity of the blood flow signals within the lesion and/or the presence of arteriovenous fistulae [45]. While hypervascularity suggests that the lesion is a hemangioma, the absence of blood flow in low-velocity flow settings suggests lymphangioma [45]. 

**Figure 13:**
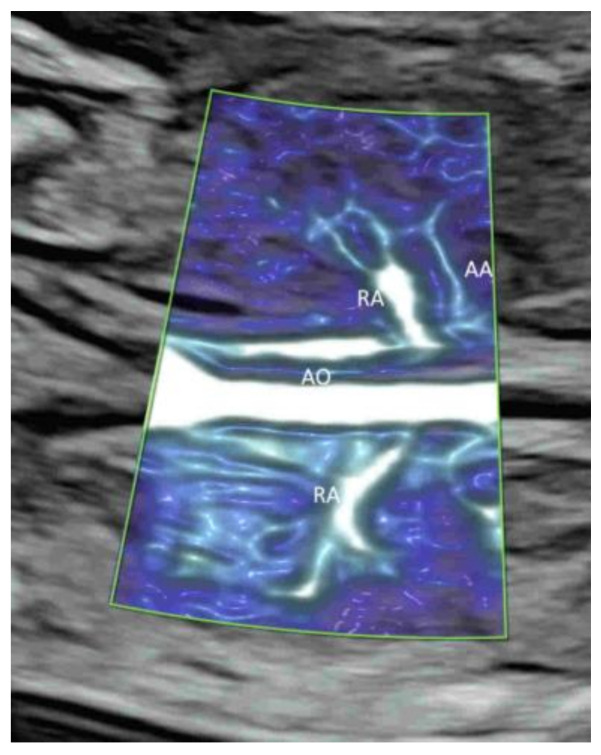
Microvascular flow imaging in a coronal plane through the back of a normal fetus at 20 weeks’ gestation showing the adrenal artery (AA). AO, aorta; RA, renal artery.

Recent studies have shown that the third-trimester size of the fetal adrenal gland is affected in fetal growth restriction or gestational diabetic mellitus [46,47]. The adrenal gland artery is well demonstrated by MVFI (Figure 13). When a cystic adrenal mass is found on prenatal ultrasonography, it is difficult to differentiate whether the mass is an adrenal hemorrhage or a cystic neuroblastoma [48]. The presence of vascularity in the outer wall or the internal septum of the mass favors the latter [49].

### 4.4. Placenta

CFI is helpful in assessing placental lakes or cysts. If there is no flow on CFI with low-flow settings, small hypoechoic vascular lakes are usually not clinically significant [50], while multiple large placental lakes are associated with fetal growth restriction [51]. The presence of multicystic lesions with varying degrees of flow within cysts using CFI with low-flow settings (stained-glass appearance) and dilated chorionic vessels are suggestive of placental mesenchymal disease, which is associated with maternal and fetal adverse outcomes [52]. Increased vascular flow in placenta lacunae and subplacental and/or uterovesical hypervascularity are features of the placenta accreta spectrum disorders (PAS) [53]. The addition of 3D ultrasound with PDI and subsequent multiplanar analysis permits an accurate assessment of the placenta–bladder interface [54]. Whether MVFI is useful in the very early stages of placental invasive abnormalities requires further studies [33]. CFI is useful in the prenatal diagnosis and monitoring of placental chorangioma with the demonstration of the feeding vessels and vascularity [55]. 

**Figure 14:**
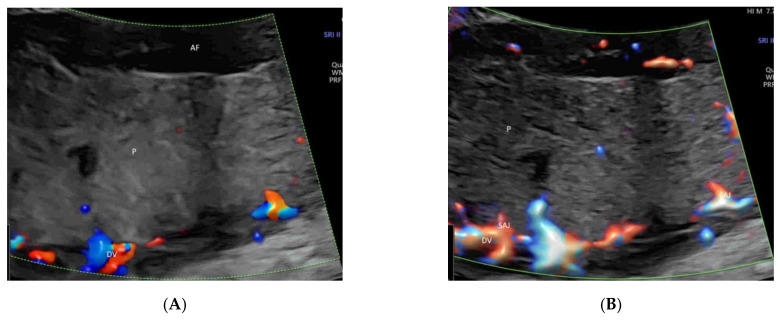

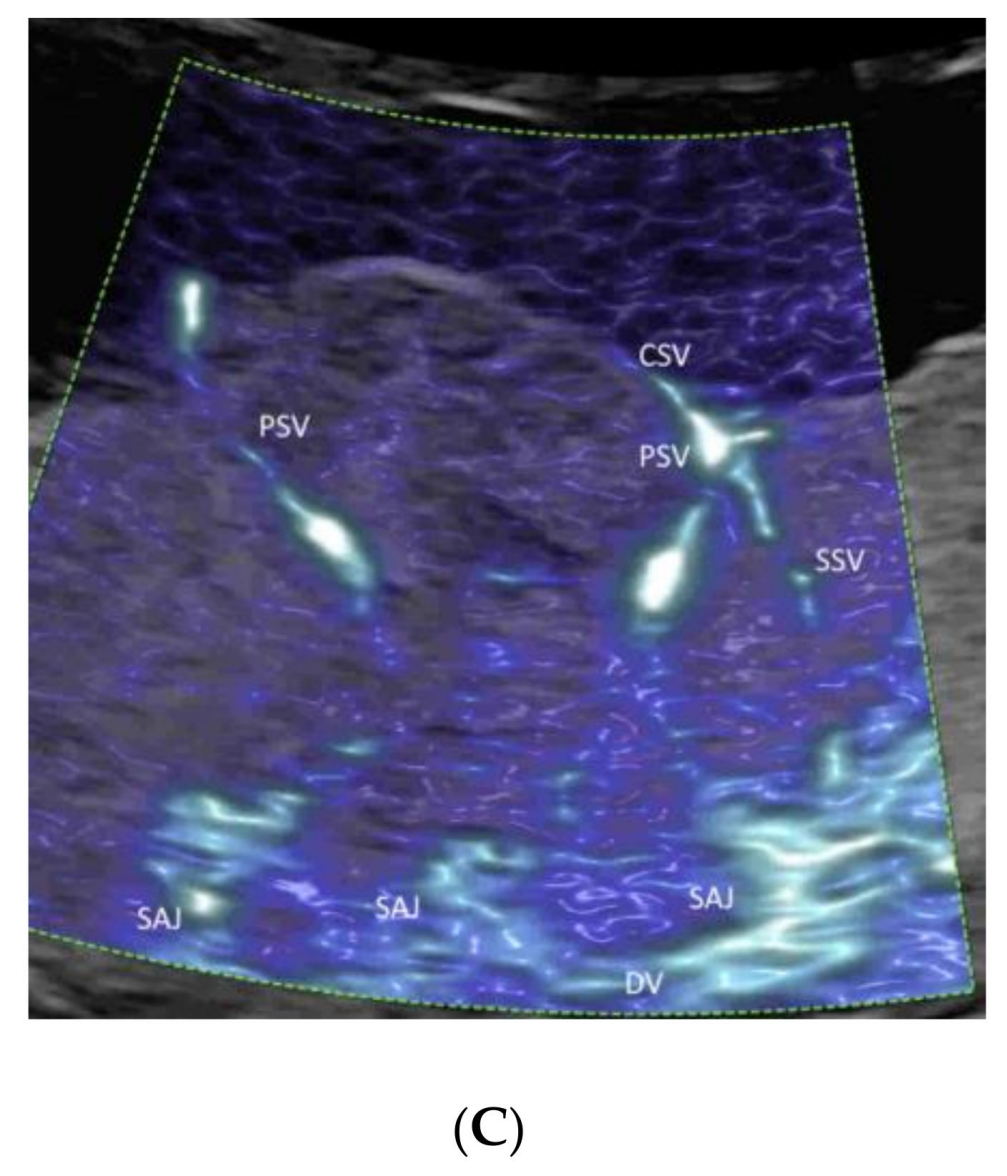
Color Doppler (**A**), high-definition (**B**) and microvascular (**C**) flow imaging of placental vessels in a normal pregnancy at 23 weeks’ gestation. Note the demonstration of the spiral artery jet (SAJ), as well as the primary (PSV) and secondary (SSV) stem villi in (**C**). DV, decidual vessels, CSV, chorionic surface vessels; AF, amniotic fluid.

MVFI provides new opportunities for noninvasive characterization of the placental microvascular pattern during pregnancy without contrast [33]. MVFI shows the intraplacental small vessels more clearly than CDI or PDI [33] while maintaining relatively high frame rates (Figure 14A–C) [56]. A recent longitudinal study has shown that it is feasible to use MVFI to assess the development of the placental microvascularization throughout normal pregnancies [57].

**Figure 15:**
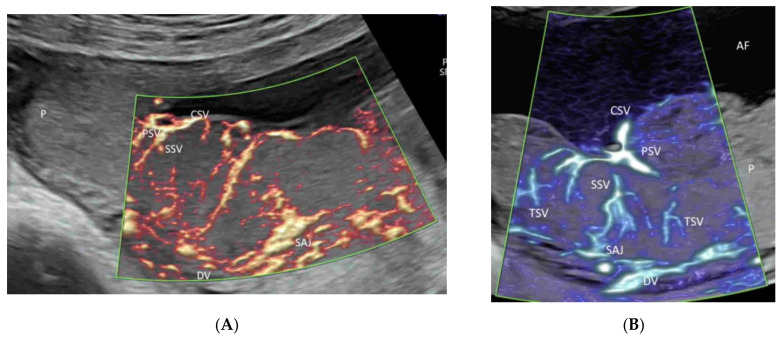

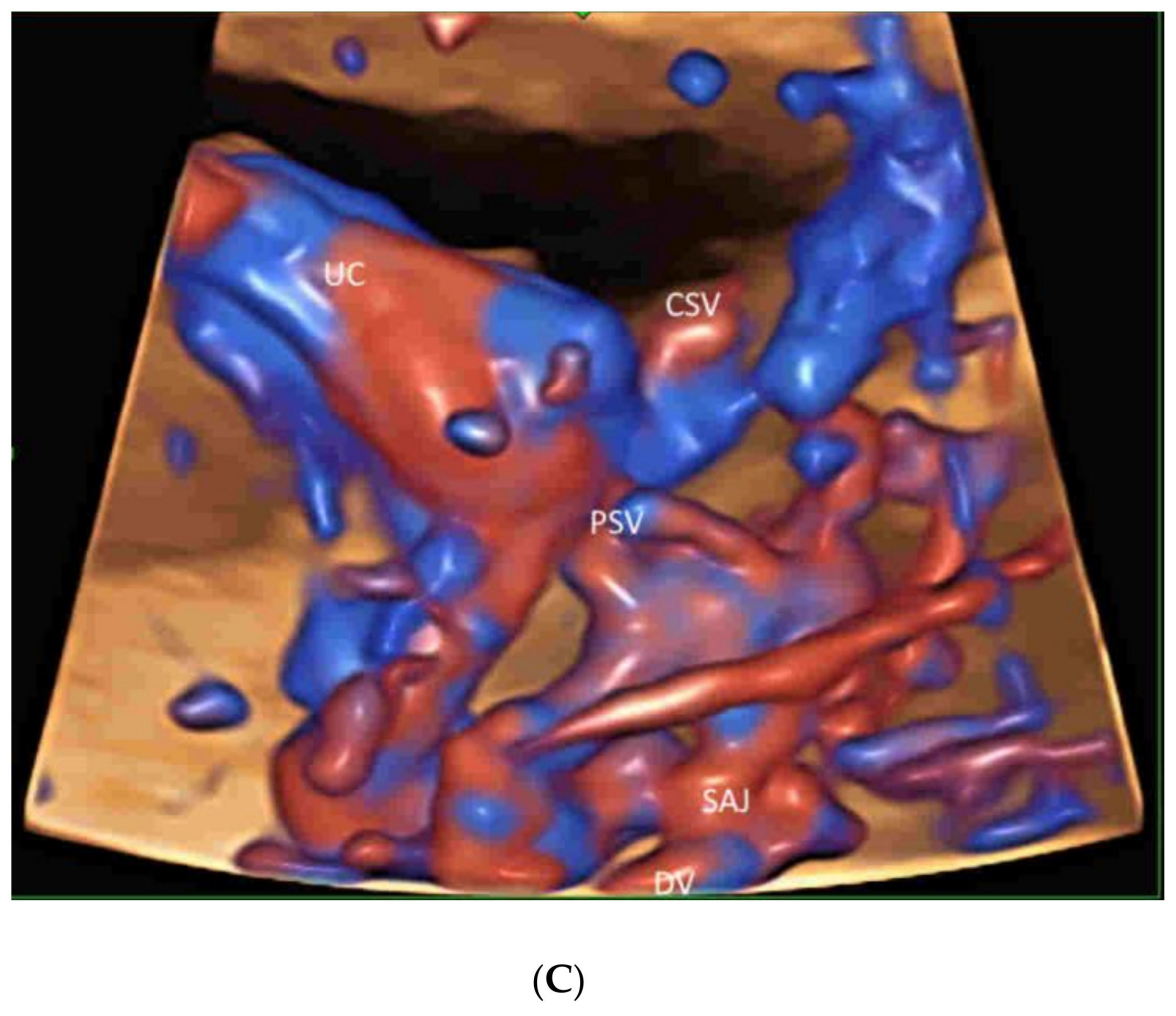
Microvascular flow imaging of placental (P) vessels in two normal pregnancies at 13 weeks’ (**A**) and at 23 weeks’ (**B**) gestation. Note the demonstration of the spiral artery jet (SAJ) and the primary (PSV) and secondary (SSV) stem villi in (**A**). In addition, the tertiary stem villi are demonstrated in (**B**). Three-dimensional ultrasound in high-definition flow displayed in the glass-body mode showing the spatial relationship of the placental vessels at 20 weeks’ gestation (**C**). Umbilical cord, UC; DV, decidual vessels, CSV, chorionic surface vessels; AF, amniotic fluid.

In the first trimester of pregnancy, MVFI can show the primary and secondary stem villous vessels and the spiral artery jet [58] (Figure 15A). In the second and third trimesters of pregnancy, MVFI can show, in addition to the above vessels, the tertiary stem villous vessels, as well (Figure 15B) [58]. A three-dimensional rendered image can show the spatial relationship of the intraplacental vessels, chorionic surface vessels, and decidual vessels (Figure 15C) [58]. 

In cases of fetal growth restriction due to placental insufficiency, MVFI shows fewer secondary and tertiary villi as compared to normal pregnancies [58,59]. Besides, primary stem villous vessels look thick and long in cases of placental infarction [58]. Whether MVFI can show an abnormal placental microvascular pattern before the development of fetal growth restriction needs further studies.

### 4.5. Umbilical Cord

**Figure 16:**
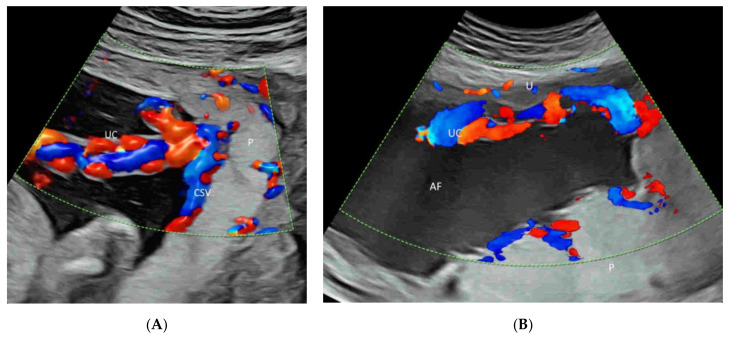
Color Doppler of the placenta showing a marginal (**A**) and velamentous (**B**) insertion of the umbilical cord (UC) to the placental (P). AF, amniotic fluid; CSV, chorionic surface vessels; U, uterus.

Identification of the cord insertion site to the placenta is facilitated using CDFI or PDI/HDFI. A velamentous or marginal insertion of the umbilical cord can be clearly seen (Figure 16A,B). CDI or PDI over the internal os may help exclude vasa previa, a critical finding [39]. 

**Figure 17:**
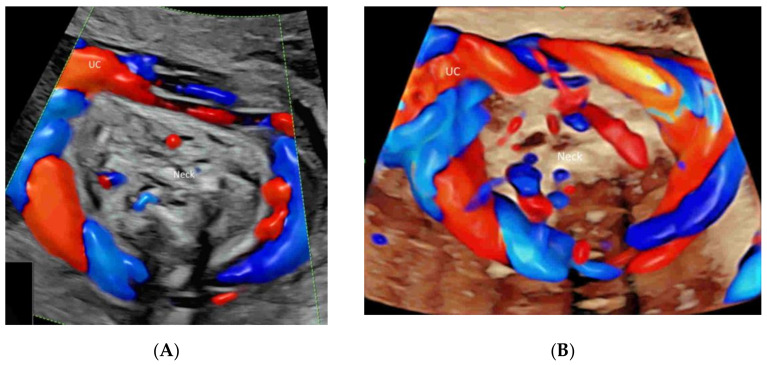
Color Doppler in a transverse plane of the neck of a fetus at 24 weeks’ gestation showing nuchal cord (**A**) with the umbilical cord (UC) wrapping around the neck. A spatiotemporal image correlation volume acquisition is displayed in glass-body mode (**B**).

CFI is useful in detecting a nuchal cord and assessing its characteristics, including the number of circles of coiling, entanglement direction, and types (Figure 17A,B) [60]. Differentiating a true knot from a false knot of the umbilical cord is not easy. A diagnosis can be facilitated by 3D/4D sonography with CDFI or HDFI [61,62].

### 4.6. First Trimester

**Figure 18:**
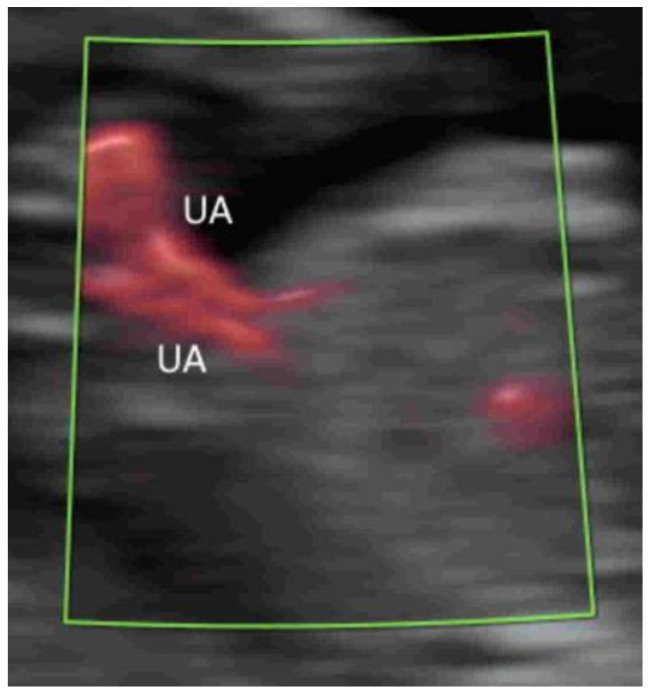
Microvascular flow imaging in a transverse plane of a fetus at 11 weeks’ gestation showing two umbilical arteries (UA).

**Figure 19:**
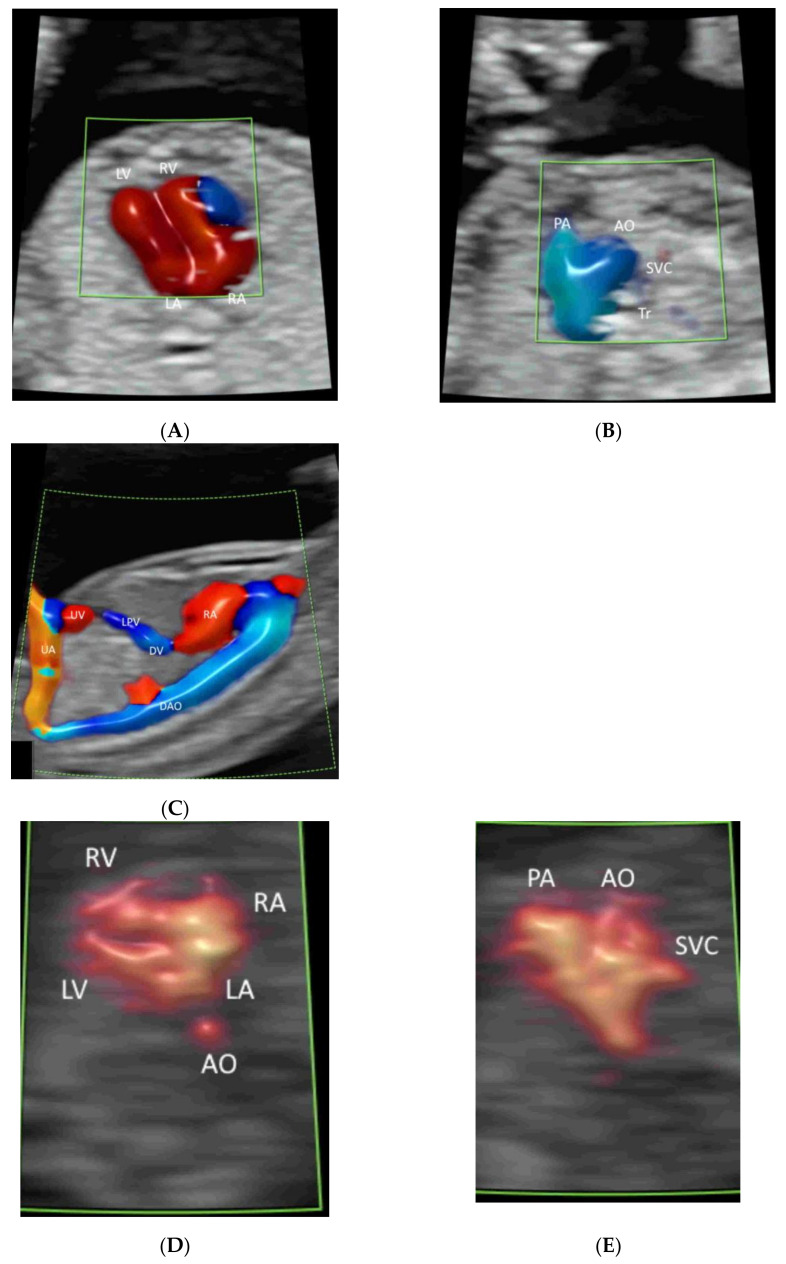
Color Doppler flow imaging with radiant flow showing the four-chamber view of the normal fetal heart (**A**), three-vessel trachea view (**B**), and ductus venosus (DV) connecting to the heart (**C**), at 12–13 weeks. Microvascular flow imaging of a normal fetal heart at 11 weeks’ gestation showing the four-chamber view (**D**) and three-vessel view (**E**). RA, right atrium; RV, right ventricle; LA, left atrium; LV, left ventricle; PA, pulmonary artery; AO, aorta; SVC, superior vena cava; Tr, trachea; LPV, left portal vein; UA, umbilical artery; UV, umbilical vein; DAO, descending aorta.

A brief evaluation of the paravesical region with CFI is helpful in confirming the presence of two umbilical arteries, though this is not part of the routine assessment (Figure 18) [8]. To achieve the early detection of major heart defects, CDFI is added to assess the four-chamber view of the heart and outflow tracts and the blood flow across the tricuspid valve and in the ductus venosus (Figure 19A–C) [63]. MVFI can enhance the resolution of the B-Mode image by showing blood flow in the four cardiac chambers (Figure 19D) and the two outflow tracts (Figure 19E) [20]. Turning on and off radiant flow can facilitate imaging of the outflow tracts and the four chambers, respectively, in the first trimester [20]. 

### 4.7. Twins

Vascular anastomoses of monochorionic placentae are the underlying cause of the development of twin–twin transfusion syndrome (TTTS) and other complications. A recent study has shown that it is feasible to use 2D and 3D ultrasonography with HDFI, combined with tomographic ultrasound imaging (TUI), to detect placental anastomoses of a monochorionic twin pregnancy [24]. Arterio-arterial anastomoses are superficial with to-and-fro blood flow signals during different portions of the cardiac cycle. Arterio-venous anastomoses are deep with a non-accompanying artery and vein branches from two twins entering the same placental lobule [24]. These findings are interesting because development of an accurate ultrasound-based mapping of placental anastomoses is an essential prerequisite for the future development of a non-invasive treatment for TTTS [24].

Twin reverse arterial perfusion (TRAP) is characterized by reverse arterial perfusion through the umbilical cord of the recipient twin, as demonstrated by CDFI or HDFI [64]. In monochorionic and monoamniotic twin pregnancies, one of the major complications is umbilical cord entanglement, which can be visualized by CDFI or HDFI.

## 5. Pitfalls of CFI

CFI are prone to errors, which are commonly caused by partial volume effects, limited temporal and velocity resolutions, inappropriate angles of insonation, aliasing, and the inability to detect slow flow [65]. Such errors may lead to misinterpretations of normal or abnormal vascular anatomy and flow. Image optimization is, thus, required, as discussed in Section 2.

Doppler artifacts can result from inappropriate machine settings, improper examination set up, reverberation from the surrounding tissue, or movement of the operator or patient. Common artifacts include random noise, aliasing, motion, and blooming. It is important to recognize, prevent, and correct these artifacts as much as possible during CFI.

Although CFI can provide flow information over a large area of interest, the information provided is limited. Volume calculation of a vein of Galen aneurysm, for instance, should be based on 3D grayscale datasets but not 3D color/power Doppler rendering [34]. The use of 3D histogram vascularity indexing, namely vascularization index, flow index, and vascularization-flow index should be cautious because of the low intra- and interobserver reliability of these indices in the assessment of placental vascularization, for instance [66]. A two-dimensional spectral Doppler examination is required to measure velocities and assess the waveform. Besides, STIC volume acquisition takes 7.5 to 12.5 s and is prone to motion artifacts. An electronic matrix transducer is an emerging technology that allows the rapid acquisition of a STIC volume with enhanced resolution [4].

## 6. Special Indications of New Modes of CFI

In pregnancies affected by or at risk of fetal growth restriction or pre-eclampsia, MVFI provides new opportunities for noninvasive characterization of the placental microvascular pattern from the first to the third trimester [33,56,57,58]. MVFI can also be used to confirm the absence of blood flow to, among others, a multicystic dysplastic kidney, an absent kidney [40], or placental lakes [50,51].

To assess abnormalities of the fetal brain, lung, liver, spleen, adrenal gland, and limbs, either HDFI or MVFI can be used to assess their vascular patterns [32,33,40]. MVFI is preferred if the assessment of small vessels or blood capillaries is required [32,33].

In cases of suspected fetal complex heart defects, using color Doppler with STIC in the glass-body mode may help the assessment of the fetal heart and great vessels [28,29,30,31]. Recently, this technique has been used to assess the abdominal precordial venous system in a cardiac cycle [38]. Adding radiant flow in CFI can improve the display of blood flow in cardiac or complex vascular structures [21].

For safety reasons, the use of the new modes as well as the conventional modes of CFI should be limited to the shortest duration possible to obtain adequate clinical information by either or both modes, in concordance with the ALARA principle [1]. At present, the new modes are not widely available. The introduction of the new modes implies additional cost but is non-invasive and less expensive compared to CT or MR angiography. A cost–benefit analysis of using the new modes over the conventional modes of CFI requires further studies.

## 7. Conclusions

Compared to previous reviews on new ultrasound technologies in obstetrics [67,68], the present review is focused on the characteristics and applications of the new as well as the conventional modes of CFI. HDFI and MVFI, which are more sensitive to low flows and small-vessel flows, are complementary to conventional CDFI. An appropriate CFI mode should be selected to study different fetal and placental circulations with various flow rates. We have highlighted specific indications of the new modes. There is an increasing use of MVFI to study the placental microvasculature. Novel or new uses include: (a) MVFI to examine the fetal heart in the first trimester, (b) MVFI to detect the early stages of PAS or placental abruption, (c) STIC with glass-body mode to examine the abdominal precordial venous system, and (d) HDFI, combined with TUI, to detect placental anastomoses of a monochorionic twin pregnancy. Further studies are required to assess their potential benefits.

## Data Availability

This review did not report any data.
